# Linear Nevus Sebaceous Syndrome in a Child With Infantile Spasms and Focal Cortical Dysplasia

**DOI:** 10.7759/cureus.17694

**Published:** 2021-09-03

**Authors:** Shahad Salman, Waseem Fathalla, Hiba Akbari

**Affiliations:** 1 Pediatrics, Sheikh Shakhbout Medical City, Abu Dhabi, ARE; 2 Pediatric Neurology, Sheikh Shakhbout Medical City, Abu Dhabi, ARE; 3 Pediatrics/Neonatal Intensive Care Unit (NICU), Addenbrooke's Hospital, Cambridge University Hospitals National Health Service (NHS) Foundation Trust, Cambridge, GBR

**Keywords:** linear nevus sebaceous syndrome, neurocutaneous syndrome, infantile spasms, cortical dysplasia, developmental delay

## Abstract

Linear nevus sebaceous syndrome (LNSS) is a rare neurocutaneous syndrome with important neurological involvement including brain malformation, focal seizures, and developmental delay. We discuss a case with a unique presentation with localization-related infantile spasms and review the clinical and radiological features of this case. To our knowledge, there are no previously reported cases of LNSS with infantile spasms and cortical dysplasia. Therefore, the presented case will make an important contribution to the available knowledge.

## Introduction

Linear nevus sebaceous syndrome (LNSS), first described in 1957, is defined by the association of a linear nevus sebaceous with cerebral, ocular, or skeletal defects [1]. It is included in the group of mosaic RASopathies, thought to be caused by sporadic postzygotic mosaic mutations in RAS genes, which are considered important components of signal transduction pathway involved in cell growth. Somatic mutation in three oncogenes, HRAS, KRAS and NRAS, has been documented in literature to be responsible for isolated nevus sebaceous [2]. The LNSS additional symptoms result from the hypothesized genetic mosaicism leading to further disrupted regulation of cell growth and division. The LNSS is characterized by sebaceous nevus. It has broad range of abnormalities including central nervous system, skeletal deformities, cardiovascular defects, urogenital defects, and an increased risk of malignancy [3]. In most cases, nevus sebaceous is evident at birth and presents as a well-defined, yellow-orange or tan, oval, round, or linear plaque. Although nevus sebaceous occurs in one in 1000 live births, LNSS is reported to occur in an estimated one in 10,000 [2]. Involvement of the central nervous system is associated with large sebaceous nevi located on the face or scalp. We report a new case of this rare neurocutaneous disorder. We aim to raise awareness about this disorder as well as emphasize the importance of early recognition of cutaneous abnormalities as part of a neurocutaneous disorder.

## Case presentation

**Figure 1:**
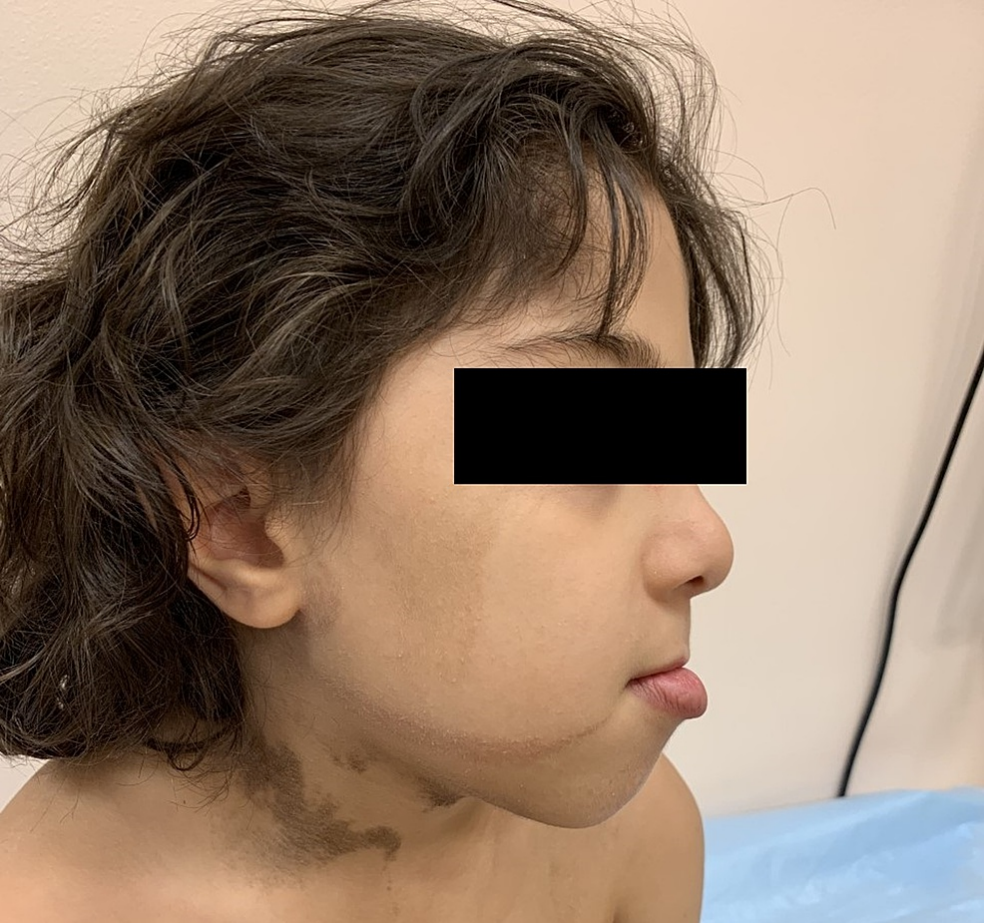
Hyperpigmented linear nevus in the right mandibular and submandibular region.

**Figure 2:**
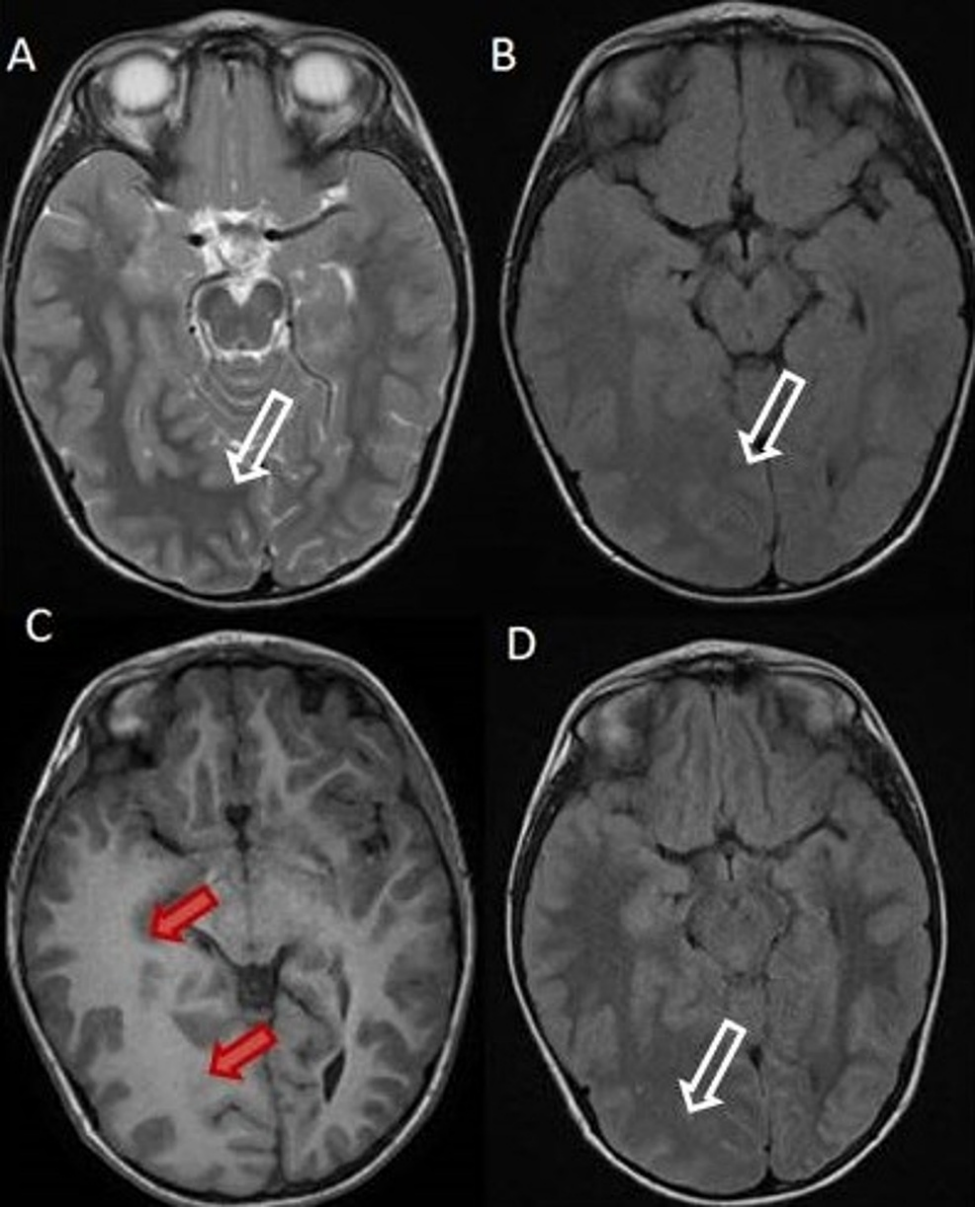
MRI brain, Axial T2 (A), Axial T1 (B), FSGR (C), and FLAIR (D) sequences showing right occipito-parietal cortical dysplasia with relatively bright and bulky white matter (red arrows) and poorly defined cortical ribbon (white arrows).

A seven-year-old boy, born to nonconsanguineous parents, presented at two months of age with clusters of flexor tonic spasms and developmental regression. His physical exam revealed a brownish hyperpigmented right-sided mandibular nevus (Figure 1), and mild esotropia. The electroencephalogram (EEG) showed “asymmetric modified hypsarrhythmia.” His spasms were fully controlled with vigabatrin with repeat EEG showing resolution of the hypsarrhythmia, but persistent right posterior focal slowing with epileptiform discharges. The MRI brain showed right posterior cortical dysplasia with mild right-sided volume asymmetry (Figure 2). Vigabatrin was stopped and oxcarbazepine was started due to high risk for focal seizures. He later developed focal aware seizures that responded to optimizing the dose of oxcarbazepine. Investigations to exclude a “forme fruste” of tuberous sclerosis complex (TSC) including cardiac echo, renal ultrasound, and genetic testing were normal. The child’s development was appropriate for age except for expressive language delay. His right mandibular/submandibular skin nevus was getting progressively darker and verruciform and clearly declared itself as a hyperpigmented linear nevus (Figure 1) ipsilateral to the focal cortical dysplasia and epileptic focus; a diagnosis of “Linear Nevus Sebaceous Syndrome” was made.

 

## Discussion

Pathology and genetics

Linear nevus sebaceous syndrome, also known as Schimmelpenning-Feuerstein-Mims syndrome (SFMS) (OMIM 163200) [4], is a rare, sporadic neurocutaneous syndrome characterized by linear sebaceous nevi that follow the lines of Blaschko, in addition to extracutaneous manifestations including the central nervous system, eyes, skeleton, and other organs [5]. Several gene mutations have been identified in the affected skin including HRAS, NRAS [6], and KRAS genes [7]. Such mutations occur postzygotically as evident by discordance between twins [7]. HRAS, NRAS, and KRAS genes are part of the RAS signaling pathway involved in cell growth regulation; postzygotic mutations result in mosaic RASopathies that are now identified in an expanding spectrum of congenital syndrome including cutaneous nevus syndrome including SFMS [8].

Clinical features

Linear nevus sebaceous syndrome is a rare congenital neurocutaneous syndrome with primary involvement of the skin (cutaneous nevi) and central nervous system (focal epilepsy, cortical malformation, and developmental delay). Extra-neurological involvement is rare.

Dysmorphology and extra-neurological manifestations

Our patient did not have any extra-neurological manifestations. The early onset of lateralization-related infantile spasms prompted investigation for possible tuberous sclerosis complex (TSC) including cardiac, ophthalmologic, and renal systems all of which were normal. His genetic testing for tuberous sclerosis was also negative. Other studies have reported extra-neurological manifestations in organs including the eyes (coloboma, strabismus), bone (cranial abnormalities, bone cysts), hypophosphatemic rickets, heart (coarctation of the aorta), kidneys (horseshoe kidney), and growth asymmetry [5, 9].

Neurological manifestations

Our patient presented with localization-related infantile spasms and regression of developmental skills that responded readily to first line medical therapy with vigabatrin but evolved into focal epilepsy with speech delay correlating with the right posterior focal cortical dysplasia. Davies et al. reported abnormal neurological findings in 7% of their cohort of 196 patients with sebaceous nevi; extensive nevi and a centrofacial location appear to be present more commonly in patients with neurological abnormalities [10]. Although apparently infrequent, the neurological involvement is the most common extracutaneous manifestation of LNSS with epilepsy, structural abnormality, and developmental delay being most common [9].

Brain imaging

More than 50% of the cases of LNSS have radiographic abnormalities, most of which affect the skull and the brain [11]. A variety of brain imaging abnormalities has been reported in LNSS including unilateral ventriculomegaly, hemimegalencephaly, hamartomas, and cortical dysplasia [11]. Our case showed unilateral occipital cortical dysplasia ipsilateral to the linear nevus correlating with the epileptic focus on EEG.

Diagnosis

A diagnosis of LNSS is usually made based on the clinical finding of sebaceous nevus along with abnormalities affecting other organ systems. Evaluation of children with LNSS should include a thorough physical exam for any growth, ocular and neurological abnormalities, and consideration for neuroimaging, echocardiography, skeletal radiography, and renal ultrasonography. Genetic testing may be considered if the distinction from other syndromes such as those mentioned above remains uncertain.

Differential diagnosis

Differential diagnosis includes other syndromes with epidermal nevi such as epidermal nevus syndrome, proteous syndrome, Cowden (PTEN) syndrome, and CLOVE syndrome [9].

Management

Management is symptomatic and is directed to the specific organ involvement. Malignant transformation within the sebaceous nevus is possible and may warrant early excision, otherwise close follow up should monitor for such transformation at which time excision is unequivocally indicated. 

Follow up

Patients should be followed up for seizure control, regular developmental assessment, and regular dermatological evaluation of the nevus for possible malignant transformation.

Prognosis

The prognosis depends on the severity of the clinical manifestations and the extent of multi-system involvement.

## Conclusions

Linear nevus sebaceous syndrome is a neurocutaneous syndrome that affects the epidermis in addition to other extracutaneous manifestations. The diagnosis of LNSS is usually made clinically, however, some may need a thorough neurological and ocular workup as well as genetic testing.

Our case highlights the importance of recognizing cutaneous abnormalities as important clues to neurocutaneous syndromes in infants presenting with focal onset seizures including infantile spasms. Once LNSS is diagnosed, a full evaluation for possible associated organ involvements is crucial and helps inform the prognosis of such children.
